# Osteoporosis and Osteopathy Markers in Patients with Mastocytosis

**DOI:** 10.4274/tjh.2013.0170

**Published:** 2015-02-15

**Authors:** Nilüfer Alpay Kanıtez, Burak Erer, Öner Doğan, Nesimi Büyükbabani, Can Baykal, Dilşad Sindel, Refik Tanakol, Akif Selim Yavuz

**Affiliations:** 1 Istanbul University İstanbul Faculty of Medicine, Department of Internal Medicine, Division of Rheumatology, İstanbul, Turkey; 2 İstanbul University İstanbul Faculty of Medicine, Division of Pathology, İstanbul, Turkey; 3 İstanbul University İstanbul Faculty of Medicine, Division of Dermatology, İstanbul, Turkey; 4 İstanbul University İstanbul Faculty of Medicine, Division of Physical Treatment and Rehabilitation, İstanbul, Turkey; 5 İstanbul University İstanbul Faculty of Medicine, Department of Internal Medicine, Division of Endocrinology, İstanbul, Turkey; 6 İstanbul University İstanbul Faculty of Medicine, Department of Internal Medicine, Division of Hematology, İstanbul, Turkey

**Keywords:** Mastocytosis, bone mineral density, Pyridinoline, bone turnover, Osteopenia

## Abstract

**Objective:**

Osteoporosis, osteosclerosis, and lytic bone lesions have been observed in patients with systemic mastocytosis (SM). We examined bone mineral density (BMD) biochemical turnover markers and serum tryptase levels in SM, which is considered a rare disease.

**Materials and Methods:**

Seventeen adult patients (5 females, 12 males; median age: 33 years, range: 20-64) with mastocytosis were included in this study. We investigated the value of quantitative ultrasound (QUS) of the calcaneus in the assessment of BMD in SM patients, as well as BMD of the lumbar spine (L1-L4), femoral neck, and distal radius using dual energy x-ray absorptiometry (DXA) and plasma tryptase levels, biochemical markers of bone turnover.

**Results:**

At lumbar spine L1-L4, the femoral neck, and the distal radius or as calcaneus stiffness, 12 of 17 patients had T-scores of less than -1 at least at 1 site, reflecting osteopenia. Three of 17 patients had T-scores showing osteoporosis (T-score <-2.5). There was no relationship between DXA and bone lesion severity. We also found a significant positive correlation between tryptase levels and disease severity, as well as between disease severity and pyridinoline (p<0.01 by Spearman’s test).

**Conclusion:**

DXA and calcaneal QUS may not be appropriate techniques to assess bone involvement in SM patients because of the effects of osteosclerosis. This study further shows that the osteoclastic marker pyridinoline is helpful in patients with severe disease activity and sclerotic bone lesions to show bone demineralization.

## Abstract (tr)

**Amaç:**

Sistemik mastositozlu (SM) hastalarda osteoporoz, osteoskleroz ve litik kemik lezyonları görülebilir. Bu çalışmada nadir bir hastalık olan SM’de, kemik yoğunluk ölçümü (BMD), serumda kemik ‘turnover’ belirteçleri ve triptaz düzeyi araştırılmıştır.

**Gereç ve Yöntemler:**

Çalışmaya 5’i kadın, 12’si erkek olmak üzere median yaşları 33 (20-64) olan toplam 17 hasta dahil edilmiştir. Hastaların BMD’leri lomber vertebra (L1-L4), femur boynu ve distal radiustan dual enerji x-ray absorbitesi (DXA) yöntemi, kalkaneustan ise kantitatif ultrasonografi (USG) yöntemi kullanılarak ayrı ayrı hesaplanmıştır. Serum triptaz düzeyleri ile biyokimyasal kemik ‘turnover’ belirteçlerileri arasındaki ilişki araştırılmıştır.

**Bulgular:**

Hastaların 12 tanesinde osteopeni (incelenen kemik alanlarının en az birinde T skoru <-1), 3 tanesinde ise osteoporoz (T skoru <-2,5) saptanmıştır. Kemik lezyonlarının şiddeti ile DXA sonuçları arasında korelasyon gösterilememiştir. Triptaz ve pridinolin seviyeleri ile hastalık şiddeti arasında pozitif korelasyon bulunmuştur (p<0,01).

**Sonuç:**

Osteosklerotik kemik lezyonları sebebiyle SM’li hastalarda kemik tutulumu değerlendirmesi için DXA ve kalkaneus USG uygun teknikler değildir. Osteoklastik aktivite belirteçleri olan pridinolin, şiddetli hastalık aktivitesi ve kemik sklerozlarını değerlendirmede faydalıdır.

## INTRODUCTION

Mastocytosis is characterized by an abnormal accumulation of mast cells (MCs) in at least one organ system [1]. MC-derived mediators and disruptive infiltration of MCs cause symptoms in mastocytosis. The clinical forms of mastocytosis are based on specific criteria such as bone marrow pathology, organ involvement, and measurement of serum tryptase [2,3]. Mastocytosis was defined in 7 categories: cutaneous mastocytosis (CM), indolent systemic mastocytosis (ISM) including bone marrow mastocytosis and smoldering systemic mastocytosis (SSM), systemic mastocytosis (SM) with associated clonal hematological non-MC-lineage disease, aggressive systemic mastocytosis (ASM), mast cell leukemia (MCL), mast cell sarcoma (MCS), and extracutaneous mastocytoma [2]. Skeletal manifestations such as osteoporosis, osteosclerosis, and lytic lesions have been observed in patients with SM, reflecting different numbers of infiltrating MCs and variation in the active substance they secrete [4]. In a recent study using dual energy X-ray absorptiometry (DXA), it was reported that SM patients with more severe disease had significantly higher bone mineral density (BMD) at the spine L1-L4 and femoral neck than patients with less severe disease [5]. However, DXA may be an inappropriate technique for assessing bone involvement in SM patients with osteosclerosis that has been observed, in addition to osteopenia in patients with SM. The purpose of this study was to assess BMD with a different technique in patients with SM and to investigate whether specific markers of mastocytosis and features of clinical disease correlate with BMD. Informed consent was obtained.

## MATERIALS AND METHODS

### Patients

Seventeen adult patients, 12 males and 5 females with mastocytosis, were included in this study. Patient evaluations included history, physical assessment, determination of plasma tryptase levels and c-kit mutation (D816V), skin biopsy, and bone marrow biopsy and aspirate. The diagnosis and classification of mastocytosis were based on WHO criteria [2]. Subgroups were defined as less severe disease (CM, ISM) and more severe disease (SSM, ASM, MCL). The subjects participating in the study had no endocrine disorders, were not menopausal women, did not receive any medication affecting the bones (including steroids and estrogens), and had no history of traumatic bone fractures. Information about risk factors of osteoporosis, including physical inactivity, tobacco-smoking habits, previous fractures, age at menarche and menopause, number of pregnancies, and consumption of milk, cheese, coffee, and medications, was obtained from individual questionnaires. According to the information obtained from the questionnaire and laboratory analysis, patients who had any risk factors for osteoporosis and those with abnormal serum parathyroid hormone (PTH) levels were excluded. In patients with low vitamin D levels, vitamin D was substituted with cholecalciferol orally and biochemical tests were done after correction of the vitamin D level. 

### Bone Mineral Measurements

BMD was measured by DXA using a Hologic QDR 4500 (Hologic, Bedford, MA, USA). Scans were performed at 3 different sites for all patients: lumbar spine (L1-L4), femoral neck, and distal radius. DXA measures the areal BMD by using ionizing radiation with photon beams at 2 different energy levels. Results were expressed as g/cm2 and T-scores representing standard deviations from young, healthy control subjects. T-scores were classified according to the WHO criteria as normal (T-scores >-1), osteopenia (-1< T-scores <-2.5) or osteoporosis (T-scores <-2.5). Simultaneously, quantitative ultrasound (QUS) measurements of the calcaneus were carried out using an Achilles Express ultrasound device (Sahara sonometer, Hologic). Calcaneal QUS involves placing ultrasound transducers on either side of the calcaneus, one acting as a wave transmitter and the other acting as the receiver. These devices measure 3 main types of parameters: broadband ultrasound attenuation (BUA), speed of sound (SOS), and the stiffness index. The stiffness index combines the parameters of SOS and BUA. Results were expressed as BMD and T-scores.

### Laboratory Measurements

Serum calcium was measured by colorimetric assay, 25-OH vitamin D by radioimmunoassay, intact PTH by chemiluminescence assay, osteocalcin by radioimmunoassay, bone-specific alkaline phosphatase (BAP) by immunoassay, and tryptase by commercial fluoroenzyme immunoassay. Pyridinoline and deoxypyridinoline were measured in morning urine samples by high-performance liquid chromatography and values were corrected for urine excretion of creatinine.

### Radiographs and Body Mass Index

Radiographs of the bones that better reflect osteopenia and/or osteosclerosis, including the spine (T4-L5), pelvis, femur, and humerus, were assessed by radiologists. Concurrently observed osteolytic and osteosclerotic lesions were interpreted as more severe bone disease than only observed osteolytic lesions. For severity of bone disease, bone radiological findings were arbitrarily documented for statistical analysis as follows: 1, normal; 2, lytic lesions; 3, severe lytic lesions and sclerotic lesions. Radiological findings were compared with BMD values. Body mass index was calculated as body weight divided by height squared.

## Statistical Analysis

Result were expressed as median (minimum-maximum). Test statistics were computed using the Mann-Whitney U test. Correlation coefficients and significance were calculated by Spearman’s test to assess the differences between groups. For all the tests, a 2-tailed p-value of <0.05 was considered statistically significant. Statistical analyses were performed using SPSS 13 on Windows NT.

## RESULTS

### Patient’s Demographics and Characteristics

The overall characteristics of the patients by diagnostic category are shown in Table 1. The age of patients ranged from 20 to 64 years of age. Five of the 17 patients were female. Four patients had CM, 9 patients had ISM, 2 patients had SSM, 1 patient had ASM, and 1 patient had MCL.

### Bone Mineral Density

The prevalence of low T-scores is shown in Table 2. Overall, 52% of patients (9 of 17 with complete data; BMD measurements at lumbar spine L1-L4, femoral neck, and distal radius and calcaneus stiffness) had T-scores between -1 and -2.5 for at least 1 site. Seventeen percent (3 of 17) had T-scores of less than -2.5. The statistical means of BMD for different areas and biochemical parameters of bone turnover and tryptase levels are shown in Table 3. The mean T-score calculated for the lumbar spine L1-L4 was lowest (-1.8±1.1) in the ISM group; 6 of 9 patients (66%) had T-scores between -1 and -2.5 and 2 patients (22%) had T-scores of less than -2.5. The mean femoral neck T-score was found to be the lowest (-0.1±0.5) in the SSM group. Two patients had femoral neck bone density of less than -1, and those patients were diagnosed with ISM. Six patients (35%) had low T-scores at the distal radius (2 with ISM and 2 with SSM). One of them with SSM had a T-score of less than -2.5. Eight patients (47%) had T-scores of calcaneus stiffness of less than -1. Significant differences for values of lateral spine BMD and all areas of radius BMD were found between female and male groups (p<0.05). Although not statistically significant, BMD of the spine and femur was increased in patients with more severe disease compared to patients with less severe disease. The results of calcaneal QUS were similar to results acquired by DXA. There was no relationship among history of hypotensive episodes, dyspepsia, diarrhea, and clinical severity. No significant correlation was found among clinical severity or serum tryptase level or any bone turnover marker and BMD calculated with not only DXA but also calcaneal QUS.

### Bone Turnover Markers

**Figure 1:**
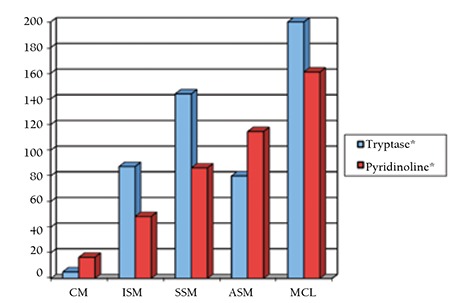
Urine pyridinoline levels (reference interval: 20-52 pmol/µmol creatinine for men, 25-63 pmol/µmol creatinine for women) and serum tryptase levels (reference interval: <13.5 µg/L) in accordance with clinical form of mastocytosis. Median values for the CM, ISM, and SSM groups containing more than 1 patient are given. *: µg/L, **: pmol/µmol creatinine.

**Figure 2:**
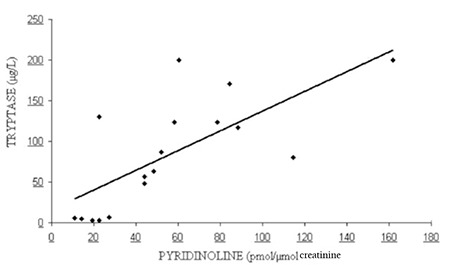
Scatter plot shows that there was a positive correlation between serum tryptase levels and urine pyridinoline levels (p<0.01).

The levels of serum ALP, tryptase, and urine pyridinoline were found to be significantly different among patient groups defined by disease severity as less severe and as more severe (respectively p<0.01, p<0.05, and p<0.01). Patients with less severe disease had lower pyridinoline and tryptase levels than patients with more severe disease (Figure 1). There was a significant positive correlation between disease severity and pyridinoline as well as tryptase levels for both sexes (p<0.01). The same correlation was shown between tryptase and pyridinoline (p<0.01) (Figure 2). Besides tryptase level, a positive correlation was found between pyridinoline and severity of radiological findings. There was no relationship between BMD and biochemical parameters of bone formations (BAP, osteocalcin, pyridinoline, deoxypyridinoline). Although a positive correlation was found between serum ALP levels and disease severity (p<0.01), there was no correlation between BAP and disease severity. It was speculated that the source of elevated serum ALP levels in patients with severe disease may be partly hepatic.

### Radiological Findings

**Figure 3:**
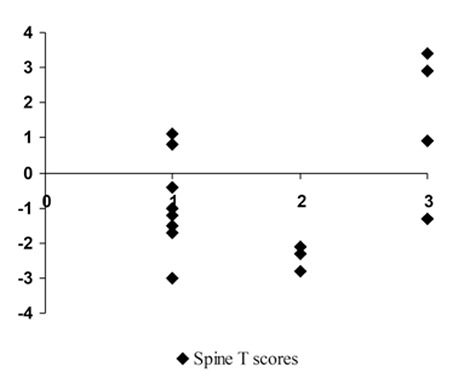
Spine T-scores of all patients are shown according to radiological severity. Radiological findings are documented as 1: normal, 2: lytic lesions, and 3: severe lytic lesions and sclerotic lesions.

No finding of bone fracture was seen on radiographs. There were lytic and sclerotic bone lesions, especially in the lumbar spine and femoral neck. Three of 17 patients (1 with SSM, 1 with ASM, and 1 with MCL) had lytic and sclerotic lesions in at least 1 site in bone radiographs. Other patients with ASM had multiple lytic bone lesions. Four of 9 patients with ISM had lytic bone lesions in at least 1 site. There were no lesions in radiographs of the patients with CM. Besides osteolytic lesions, patients with severe disease had more osteosclerotic lesions on their radiographs than patients with less severe disease. The relationships among T-scores of the spine and femoral neck and radiological findings of those bone areas for each patient are shown in Figure 3. The patients with less severe disease had lower T-scores compared to patients with more severe disease. Considering radiological findings, the reason for this is the increased osteosclerosis in more severe disease.

## DISCUSSION

The frequency of bone changes varies with the clinical form of SM. The most common radiological findings associated with SM consist of concurrent osteosclerotic and osteolytic lesions (45%) [2]. According to previous studies, trabecular and cortical bone turnover increases in the regions of MC accumulation [6]. In trabecular or cortical bone, accelerated bone remodeling is remarkable, with expansive peritrabecular fibrosis, osteoidosis, increased numbers of osteoblasts and osteoclasts, and extension of osteoclastic resorbing surfaces [7]. Excess MCs are associated with accelerated bone loss and remodeling states. Heterogeneous groups of mediators such as the granule-associated mediators (histamine, heparin, and neutral proteases) and lipid-derived mediators (lipoxygenase, cyclooxygenase, leukotrienes, and prostaglandin D2) are released from MCs [8]. Histamine, the most important mediator produced and stored, has been shown to modulate osteoclastic activity. Thereby, the MCs may lead to osteoclastic bone resorption by increasing osteoclasts [9]. Large-sized osteolyses or/and severe osteoporosis causing pathologic fractures is one of the C findings defining disease stage and severity [2,5].

Radiographic methods, including roentgenography, magnetic resonance imaging, nuclear bone scan, and bone densitometry, have been employed to assess the patterns and severity of skeletal involvement [10,11]. DXA is currently the most frequently used instrument for measuring BMD [12]. Previous studies showed that DXA is unable to detect changes in trabecular bone microstructure in relation to changes in its mechanical properties [13]. Although DXA is a useful technique to detect bone mineral density in patients with osteolytic lesions, patients with severe disease had more sclerotic changes, especially in the trabecular bone of the pelvis and the thoracolumbar spine. Those patients had an increased level of bone density in trabecular bone and low bone density in the distal radius. There was not a statistically significant correlation between BMD of any area and disease severity. DXA is probably not adequate to detect bone changes in patients with aggressive mastocytosis because osteosclerotic lesions may occur concurrently and cause higher BMD scores despite the occurrence of osteopenia [4,5].

There is increased interest in calcaneal QUS for osteoporosis screening as an additional method because it predicts fracture risk, is portable, and is relatively inexpensive [14]. Ultrasound parameter values are typically lower in osteoporotic bone than in healthy bone [15,16]. On the other hand, there is no consensus regarding its accuracy for identifying patients with osteoporosis, and osteosclerosis is also a problem in screening for osteoporosis in patients with mastocytosis, as in our results [17]. Therefore, calcaneal QUS does not seem to be a useful method compared to DXA in estimating bone involvement in patients with mastocytosis.

Direct radiography was found to be more useful for showing bone involvement in our patients. Both osteosclerotic and osteolytic lesions can be shown on radiographs, especially in severe disease. Although there were no findings of fracture in the radiographies of our patients, pathological fractures may be seen in mastocytosis patients [18,19].

Biochemical markers of bone turnover, such as pyridinoline and BAP, may increase in other diseases, such as hyperthyroidism, suggesting an increase in osteoclastic and osteoblastic activity [20]. Johansson et al. found that the level of serum ALP was significantly higher in mastocytosis patients with severe osteoporosis [4]. However, BAP is a more specific marker because serum ALP may be elevated in severe cases with hepatic involvement. We used BAP, osteocalcin, pyridinoline, and deoxypyridinoline as bone turnover markers and observed that patients with severe skeletal lesions had greater increases in levels of BAP and pyridinoline than patients with mild skeletal lesions. The relationship between BAP and disease severity was not statistically significant, whereas there was a significant correlation between pyridinoline and disease severity (p<0.01).

Kushnir-Sukhov et al. showed that elevated tryptase levels were associated with greater bone density in patients with mastocytosis [5]. We could not confirm this relationship in this study. Both our study and the study done by Kushnir-Sukhov (21 patients) had a small number of patients because mastocytosis is a very rare disease. Tryptase is a well-established and important disease-related marker that should be determined in patients with suspected mastocytosis [21,22]. Higher tryptase values increase the likelihood of multiorgan involvement. Moreover, tryptase levels in SM are thought to correlate with the burden of neoplastic MCs [23]. In our study, there was a similar correlation between disease severity and serum tryptase and urine pyridinoline; patients with severe bone lesions had a greater elevation of pyridinoline levels. According to a previous study, tryptase may activate matrix metalloproteinases (MMPs) [24]. MMP can degrade collagens and may increase the pyridinoline cross-linked carboxyterminal telopeptide of type 1 collagen. Therefore, it may be speculated that one cause of osteolytic bone lesions may be tryptase associated with elevated pyridinoline levels; however, this hypothesis is insufficient to explain osteosclerosis in more severe disease.

In conclusion, this study confirmed that SM can affect bone remodeling in various ways and demonstrated the need for radiography in bone evaluation. BMD measured by DXA or calcaneal QUS may not be decreased in patients with osteopenia due to osteosclerotic bone lesions seen on plain radiography. In follow-up, increase in serial BMD measurements related to the osteosclerotic lesions in patients with SM may be used to determine the progression of bone involvement. High pyridinoline levels may indicate high bone turnover in mastocytosis and are associated with more severe bone lesions. However, it is not clear whether high pyridinoline levels are the result of elevated tryptase levels. The number of patients studied here is rather small; thus, further work is needed.

## Figures and Tables

**Table 1:**
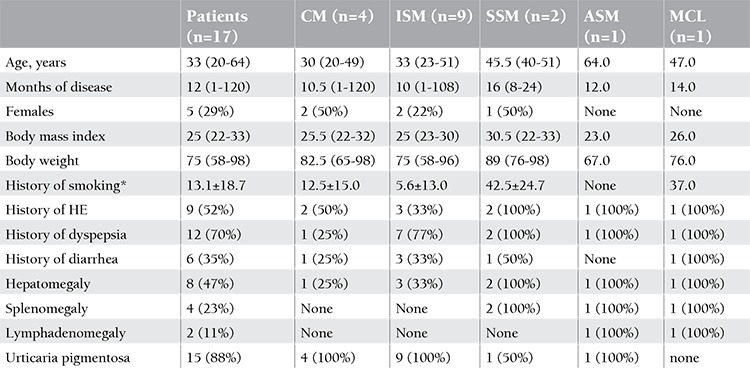
General characteristics of patients with mastocytosis.

**Table 2:**
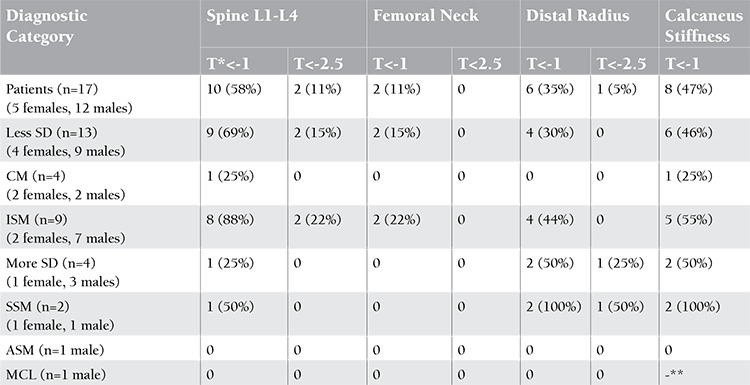
T-scores of the lumbar spine, femoral neck, distal radius, and QUS of the calcaneus for each mastocytosis category. T-scores calculated with DXA were obtained for all patients but calcaneal QUS could not performed for 1 patient because there was anatomical mismatch between the device and the patient’s heel.

**Table 3:**
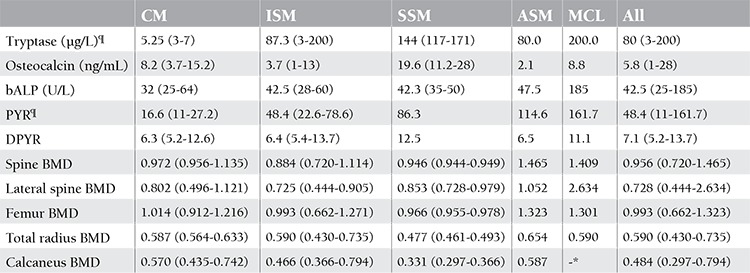
Biochemical parameters and BMD of spine, femoral neck, total radius, and calcaneus for each mastocytosis category.
